# Phenylketonuria (PKU): A problem solved?

**DOI:** 10.1016/j.ymgmr.2015.12.004

**Published:** 2015-12-29

**Authors:** Christine S. Brown, Uta Lichter-Konecki

**Affiliations:** a National PKU Alliance, Tomahawk, WI 54487, United States; b Section of Neurology, St. Christopher's Hospital for Children, Dept. of Pediatrics, Drexel University College of Medicine, United States

**Keywords:** ACMG, American College of Medical Genetics and Genomics, NPKUA, National PKU Alliance, PAH, phenylalanine hydroxylase, Phe, phenylalanine, PKU, phenylketonuria, Phenylalanine, Phenylketonuria, Phenylalanine hydroxylase deficiency

## Abstract

Phenylketonuria (PKU) is a rare metabolic disorder characterized by impaired conversion of phenylalanine (Phe) to tyrosine. If left untreated, the resultant accumulation of excess blood Phe can cause physiological, neurological, and intellectual disabilities. The National PKU Alliance (NPKUA) conducted a survey of its membership to assess current health status and interest in new treatments for PKU. Of the 625 survey respondents, less than half (46.7%) reported blood Phe within (120–360 μmol/L) — the range recommended by the American College of Medical Genetics and Genomics (ACMG). The survey results also showed that younger (≤ 18 years) individuals were about 3-times as successful in keeping their blood Phe concentrations within the recommended clinical range compared with adults. Blood Phe over 360 μmol/L was reported in one-quarter (25.5%) of ≤ 18 year old individuals and almost two-thirds (61.5%) of adults. A little more than half (51.7%) of respondents reported having difficulty in managing their PKU, including the maintenance of a Phe-restricted diet. Individuals with PKU desire new treatments that would allow them to increase their intake of natural protein, discontinue or reduce their intake of medical foods (medical formula and foods modified to be low in protein), improve their mental health (including a reduction in depression and anxiety), and a reduction of their blood Phe concentrations. Respondents preferred oral administration of any newly developed therapies and, in general, disliked therapeutic injections. Injections at home were preferred over injections at a clinic. Payers, government agencies, clinicians, and industry partners should consider patient input when developing and approving new therapies and treatments for PKU.

## 1 Introduction

Phenylketonuria [PKU, MIM 261600, also referred to as phenylalanine hydroxylase (PAH; EC 1.14.16.1) deficiency] is a rare (prevalence < 1/10,000–1/15,000 births) autosomal recessive disorder characterized by an impairment of the body's ability to metabolize phenylalanine (Phe) [1]. Without a fully functional PAH enzyme, Phe accumulates in the blood, brain, and body tissues. High concentrations of blood Phe are toxic to the central nervous system and can cause severe neurological complications and intellectual disability [2]. The new treatment guidelines of the American College of Medical Genetics and Genomics (ACMG) state that the clinical treatment goal for individuals with PKU are to maintain blood Phe within the range of (120–360 μmol/L) for individuals of all ages and that treatment should be lifelong [2].

Newborn screening for PKU was instituted in the 1960s to prevent the most serious consequences of the disease [2], [3]. Dietary treatment [including the use of Phe-free medical foods and the avoidance of high protein foods] successfully lowers blood Phe in most individuals with PKU [4]. Pharmaceutical treatments include the use of sapropterin dihydrochloride (a synthetic form of tetrahydrobiopterin (BH4) — the natural co-factor for the PAH enzyme). Studies have shown that sapropterin dihydrochloride is well tolerated, lowers blood Phe, and improves Phe tolerance in approximately 25–50% of individuals with PKU [2], [5].

There are substantial unmet needs of individuals with PKU [6]. Lifetime adherence to a Phe-restricted diet is challenging and blood Phe is difficult to maintain within the recommended range. Even individuals who successfully manage their blood Phe from birth can exhibit subtle deficits in neuropsychological functioning [7], anxiety, depression, and executive function deficits can occur even in those individuals who are well controlled [2]. Therefore, new therapies are needed to improve the quality of life of individuals with PKU across their lifespans [8].

The National PKU Alliance (NPKUA) is a patient advocacy nonprofit organization dedicated to improving the lives of individuals with PKU and assisting in the development of a cure. Formed in 2008 by 17 state and regional support groups across the country, the NPKUA focuses on critical and unmet needs of the PKU patient community. Patient advocacy organizations (like NPKUA) are important because they disseminate reliable information about new treatments to affected individuals and their families [9]. The NPKUA provides information and support to adults and families of children with PKU, advocates for the reimbursement of Phe-free and modified low-protein foods, and invests in targeted and peer reviewed research for the development of new therapies and a future cure for PKU.

The NPKUA conducted a survey of adults and children with PKU in the United States to better understand the current and future needs of the PKU community. The goal of the survey was to help inform the NPKUA's research priorities, provide feedback from individuals to medical providers, payers, and other stakeholders, and to serve as a resource to industry to ensure the patient perspective is central to the development of new therapies.

## 2 Methods

The NPKUA conducted a self-selected, non-randomized patient survey during May–June 2015 that focused on current therapy status, treatment preferences, and risk tolerance for new treatments. The survey consisted of 28 questions, 21 primary questions and 7 supplemental questions that asked for either categorical, free form, or Likert-scale responses (Table 1). A link to an online survey was distributed to NPKUA members and families via e-mail, posted on the PKU listserv, posted on the NPKUA's Facebook page, and sent out via e-mail to addresses collected through the PKU.com website. If more than one child had PKU in a household, parents and caregivers were asked to respond to the survey using their experience of the oldest child with PKU.

### 2.1 Statistical analysis

Response rates were computed using the number of respondents to each survey question. Some responses required the responder to self-report their data according to interval ranges, other responses used Likert-Scales, and some responses permitted free-form entries. Similar Likert-Scale responses were combined to simplify the statistics (e.g., ‘very easy’, ‘easy’, and ‘somewhat easy’ responses were combined into a single group). Information regarding gender, BMI, and highest lifetime Phe was not requested. An ANOVA test was used to compare group means using 95% confidence intervals.

## 3 Results

### 3.1 Survey response

From a pool of 6312 potential respondents, at total of 625 (9.9%) responded to the survey. Respondents included individuals with PKU (N = 220), parents of children with PKU (N = 362), other caregivers (N = 39), and others (N = 4). Of the 21 primary questions, 19 had a response rate of 74% (N = 465) or higher and 13 questions had a response rate of 96% (N = 606) or higher. Respondents were almost evenly divided by age. About half of respondents (53%, 332/622) were ≤ 18 and half (47%, 290/622 were > 18 years of age.

### 3.2 Current PKU clinical status

**Fig. 1:**
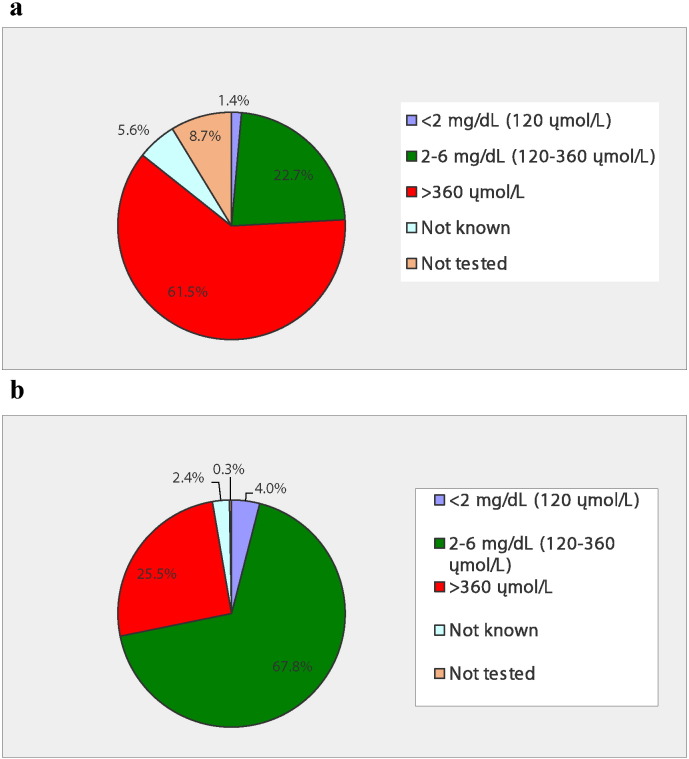
a, b. Percentage of blood Phe values within the range of < 120, 120–360, and > 360 μmol/L for individuals with PKU > 18 years (Fig. 1a, N = 286) and ≤ 18 years (Fig. 1b, N = 329) during the past year.

Over half (61.8%, 383/620) of those surveyed said that they had their blood drawn to monitor blood Phe concentrations in the last month. More than two-thirds (68.4%, N = 423) stated that they desired blood Phe within the ACMG range of 120–360 μmol/L. However, less than half (46.7%; 288/617) managed to attain Phe within this range. There was a difference in Phe control by age. Fig. 1 shows that individuals with PKU ≤ 18 years were 3-times as likely to have blood Phe within the 120–360 μmol/L range compared to adults > 18 years. Fig. 1 also shows that Phe concentrations > 360 μmol/L were reported for one-quarter of ≤ 18 year old individuals and almost two-thirds of > 18 year old individuals. For individuals with blood Phe concentrations > 360 μmol/L, the mean for ≤ 18 year old individuals (447.6 ± 116.5 μmol/L, N = 63) was not significantly different compared to > 18 year old individuals (477.0 ± 120.3 μmol/L, N = 160).

### 3.3 Current PKU therapy

Overall, 70% of survey respondents reported consumption of medical formula, 54% consumed modified low-protein foods, 76% consumed foods that are naturally low in protein, 40% used sapropterin dihydrochloride, 3% were prescribed large neutral amino acids (LNAA), while 6% stated they are currently not treating PKU with medical foods or pharmacotherapy. Younger individuals reported consuming medical foods more than adults. Sapropterin dihydrochloride use was not associated with subject age.

Fifty-eight percent of individuals with blood Phe < 360 μmol/L reported clinic visits within the past year compared to 38.6% of individuals with blood Phe > 360 μmol/L. Eight-percent of respondents reported that it had been more than two years since their last clinic visit. Individuals on sapropterin dihydrochloride therapy generally had more recent metabolic clinic visits; however, most individuals (86.3%) reported visiting a metabolic clinic within the past year. About two-thirds (68%) of respondents said that they had followed their PKU treatment plan for all or most of their life.

**Fig. 2:**
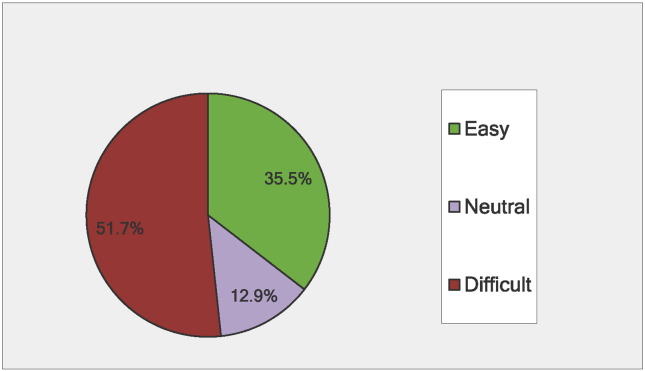
Percentage of respondents reporting that managing PKU treatment was: Easy^1^, Neutral, or Difficult^2^ (N = 615). 1: Combines Likert scale responses for: ‘very easy’, ‘easy’, and ‘somewhat easy’ 2: Combines Likert scale responses for: ‘somewhat difficult’, ‘difficult’, and ‘very difficult’.

Fig. 2 shows that about a third of respondents reported that managing their PKU was easy, while half reported that PKU management was difficult. Similarly, two-thirds reported that PKU restricted their lifestyle. More adults reported struggling with their treatment plan than children. Almost half (49.5%) of individuals with blood Phe within the recommended range reported that their PKU was easy to manage compared to 20.7% of individuals with blood Phe > 360 μmol/L. More individuals on sapropterin dihydrochloride reported that their PKU was easy to manage when compared to individuals on other treatments (p < .0001) and sapropterin dihydrochloride use was associated with lower blood Phe (p < .0001).

### 3.4 Future therapy and treatments

When asked about the importance of new treatments, 474 individuals responded. A majority (91.4%, N = 433) of those responding stated that it was ‘somewhat important’, ‘important’, or ‘very important’ to develop new treatments for PKU, 4.2% (N = 20) were neutral on the topic, and 4.5% (N = 21) stated that new treatments were not important. When the responses were subdivided by subject blood Phe the desire for new treatment did not depend on the concentration of blood Phe. The majority (92.7%, 202/218) of individuals with blood Phe between 120 and 360 μmol/L stated that new treatments were important as did 89.9% (187/208) of individuals with blood Phe > 360 μmol/L.

Respondents were asked to select a statement that best reflected their feeling about their current PKU treatment plan and their hopes for the future. Over half (57%) responded that they were happy with their current treatment plan but hoped that new treatments would be available in the future. About a third (35%) stated that they struggle with the current treatment plan or have no current treatment plan but also hope for new treatments.

Table 2 shows the survey results when respondents were asked which symptoms of PKU they would like to improve as a benefit of new treatments. A total of 463 individuals responded and reported that blood Phe reduction, improved attention, and improved executive function were the most important potential benefits of new treatments. Results were consistent when the responses were subdivided by subject blood Phe. Eighty-two (82%, 169/206) percent of individuals with blood Phe 120–360 μmol/L desired treatments that had the potential to lower blood Phe whereas 95% (200/211) of individuals with blood Phe > 360 μmol/L desired this same benefit.

Table 3 shows respondent preferences regarding which lifestyle improvements they considered most desirable in the context of future treatments — and over three-fourths (77.7%) desire a treatment that allows for an increase in natural protein intake without an increase in symptoms.

### 3.5 Benefit/risks and adverse events related to new treatments

Respondents were asked to describe their interest in therapeutic administration of new therapies using a 7-point Likert scale (from ‘not at all interested’ to ‘extremely interested’). Table 4 shows that respondents prefer oral ingestion for new therapies and dislike injections. Injections at home were preferred over injections at a medical facility — and half stated no interest in daily injections at a medical facility.

Table 5 shows the results of willingness to tolerate at least some temporary injection site pain (81%) or mild skin irritation (79%). However, internal bleeding and severe allergic reactions were generally considered intolerable side effects. Two-thirds (66.8%) were willing to tolerate immunosuppressant drugs for ≤ 6 months, 56.3% for ≤ 1 year, 29.7% for ≤ 5 years, and 22.6% for ≤ 10 years.

## 4 Discussion

With the advent of newborn screening and the development of medical foods and dietary therapy, severe mental retardation due to PKU has abated. This progress has resulted in significant economic gains to society as well as improvements in quality of life and health benefits for affected individuals [2]. Many clinicians and health care organizations may hold the view that, for individuals with PKU, current strategies and therapies are therefore adequate and further healthcare investment is no longer needed.

In contrast to this view, these NPKUA survey data show that many individuals with PKU report difficultly managing their disease, have problems maintaining their blood Phe concentrations within the recommended range, and that the ability to control blood Phe worsens with age. Managing PKU is difficult, complex, and life-long. There are substantial challenges for individuals with PKU including compliance with a Phe-restricted diet, limited therapeutic options, side effects of existing treatments, lack of insurance coverage for medical foods, non-reimbursed costs, and a need for palatable alternatives [6], [10]. Adults with PKU face even more challenges and comorbidities [11].

The survey data confirm that dietary therapy remains the mainstay of clinical treatment with three-quarters of the cohort consuming natural low-protein foods. More individuals on sapropterin dihydrochloride reported that their PKU was easier to manage when compared to those not receiving sapropterin dihydrochloride (p < .0001) and sapropterin dihydrochloride use was associated with lower blood Phe (p < .0001). However, responsiveness to sapropterin dihydrochloride varies from 25 to 50% of individuals with PKU [2]. The majority of surveyed individuals stated that development of new treatments and therapies is an important goal. Table 2 shows that the most desired outcomes for new treatments would reduce blood Phe concentrations, improve attention, and improve executive function. Respondents preferred oral administration of any newly developed therapies with desired outcomes of a lower blood Phe and improvement in neuropsychological performance. Injections at home were preferred over injections at a medical facility — half (50.1%) have no interest in daily injections at a medical facility. This information may be pertinent to current trials of rAvPAL-PEG, which is administered by injections with concomitant mild to moderate skin and injection site adverse events [6].

Blood Phe is a compliance measure for PKU clinical treatment as well as a biomarker of disease severity. Only about half of survey respondents reported blood Phe within 120–360 μmol/L – the ACMG recommended range – even though most were knowledgeable about this guidance. Fig. 1 shows that younger (≤ 18 years) individuals were 3-times as successful in keeping their blood Phe concentrations within the recommended clinical range compared with adults. High blood Phe was reported more often for adults and less often in children and adolescents. These data confirm previous studies that difficulty managing PKU increases with age [12], [13], [14]. In early childhood, PKU dietary compliance is often high due to high parental control and low peer pressure; however dietary compliance generally decreases in adolescence and adulthood [15]. Reinstatement of dietary therapy after discontinuation in adolescence or adulthood can be extremely difficult [16]. In adults, lack of insurance coverage for medical foods the temptation of eating high protein foods once the patient has developed a taste for them, insufficient knowledge of PKU, and socio-economic status are likely influences for adherence to the very difficult PKU diet.

These survey data have several limitations — most of which are typical of survey questionnaires. The survey data were weighted toward informant-report and had less representation of either self-report or caregiver report. While data were not clinically verified and medical histories were unknown, the blood Phe data are consistent with previous studies [12], [13], [14]. The NPKUA questionnaire was not assessed for reliability or bias (including social desirability bias). Many of the survey questions asked for opinions and not verifiable facts. However, none of the conclusions conflict with published evidence. Survey responders may have been more motivated and compliant than other individuals, which may explain the high rate (86%) of annual clinic visits in this cohort compared to a previous report [17] as well as the relatively low mean blood Phe even for the group of individuals with blood Phe above 360 μmol/L.

## 5 Conclusions

Individuals with PKU face a variety of challenges and unmet needs. Even individuals who are conscientious with self-care, and who have adequate Phe control as a result, report difficulties treating their condition and maintaining recommended treatment. The NPKUA survey data show that many individuals with PKU report difficultly managing their disease, have problems maintaining their blood Phe within the recommended range, and that the ability to control blood Phe worsens with age. In addition, many individuals with PKU desire new therapies and treatments with tolerable side effects. Payers, government agencies, clinicians, and industry partners should consider patient input when developing and approving new therapies and treatments for PKU.

## Potential conflicts of interest & competing interests

Christine S. Brown is a parent of children with PKU. The National PKU Alliance received funding from BioMarin Pharmaceutical Inc. to conduct the statistical analysis and draft the manuscript. Uta Lichter-Konecki received travel support from BioMarin to serve as an advisor in a PKU/Phenylalanine Hydroxylase (PAH) Deficiency Treatment Guidelines Consultancy Meeting in July 2014; no other conflicts.

## Figures and Tables

**Table 1:** National PKU Alliance (NPKUA) survey questions and question type.

Number	Question	Response type
Q01	What is your relationship to PKU?	Categorical
Q02	How old are you when completing this form?	Categorical
Q03	Select the statements below that reflect how you currently treat your/your loved one's PKU	Categorical
Q04	When was the last time you drew blood to monitor Phe levels?	Categorical
Q05	What has been the average blood Phe level in the last years?	Categorical
Q06	Select preferred blood Phe level	Categorical
Q07	When was the last visit to a metabolic clinic that focuses on PKU patients?	Categorical
Q08	Select the statement below that best reflects your current PKU treatment status.	Categorical
Q08x	Explain ‘Other’ current PKU treatment status	Categorical
Q09	Indicate difficulty managing PKU	Likert
Q09x	Explain ‘Other’ difficulty managing PKU	Free form
Q10	Indicate how PKU restricts lifestyle	Likert
Q10x	Indicate how PKU restricts lifestyle	Free form
Q11	Select statement that best reflects current PKU treatment plan	Categorical
Q12	Indicate importance of having new PKU treatments	Likert
Q13	What benefits do you hope to see with a new PKU treatment?	Free form
Q14	What symptoms or results would you like to see with a new PKU treatment?	Categorical
Q14x	Explain ‘Other’ symptoms or results with new treatment	Free form
Q15	Rank the symptoms or results from Question 14	Likert
Q15x	Force Rank the symptoms or results from Question 14	Likert
Q16	What change in lifestyle would you like to see having a new PKU treatment?	Categorical
Q16x	Explain ‘Other’ change in lifestyle	Free form
Q17	Rank the two top changes in lifestyle from Question 16	Likert
Q17x	Force Rank the two top changes in lifestyle from Question 16	Likert
Q18	Indicate willingness to new PKU treatments	Likert
Q19	Indicate willingness to tolerate side effects from a new treatment	Likert
Q20	Indicate willingness to take immunosuppressant each time period	Likert
Q21	Please tell us anything else you would consider in deciding to choose a new therapy for PKU	Free form

x: Questions with an ‘x’ relate to the immediately preceding question and denote either a corollary question or a request for more information.

**Table 2:** Ranked responses for most desired outcomes when considering new treatments.

Desired individual preferences	Response percent	Response count
Drop in blood Phe concentrations	87.5%	405
Improved attention span and ability to focus	65.7%	304
Improved executive function skills, such as the ability to plan, organize and prioritize	61.6%	285
Reduced depression, anxiety and/or ups and downs in overall mood	55.1%	255
Improved processing speed — the ability to start and complete tasks	52.1%	241
Increase in energy	51.0%	236
Improved memory	49.5%	229
Lifting of “the fog”	43.0%	199
Reduced bone loss	30.0%	139
Reduced tremors	19.2%	89
Reduced other damage such as muscle weakness, and gait disorders	18.8%	87

**Table 3:** Ranked responses when asked which lifestyle improvements were most desired.

Preferences	Response percent	Response count
I would like to be able to increase my protein intake without increasing my symptoms of PKU.	77.7%	365
I would like to be able to eat any foods I choose regardless of their protein content.	76.0%	357
I would like to be able to consume less of my medical foods (formula and low protein foods).	57.7%	271
I would like to discontinue the use of my medical foods (formula and low protein foods).	47.2%	222
I would like to have better mental health.	45.7%	215
I would like to improve my social relationships.	34.5%	162
I would like to decrease the frequency of blood tests.	28.9%	136

**Table 4:** Ranked Responses when Asked their Degree of Interest in the Method of Administration of New Therapies.

Methods of therapeutic administration	No interest (%)	Neutral (%)	Some or strong interest (%)
Weekly consumption of a probiotic	1.7	7.2	91.1
Daily oral pill(s)	3.9	7.6	88.5
Daily consumption of a probiotic	2.8	9.5	87.7
Monthly injection at home	9.3	4.8	85.9
Inserting a gene into your cells at a medical facility to correct PKU	7.3	7.1	85.7
Infusion of stem cells over several days at a medical facility to correct PKU	8.1	9.2	82.7
Weekly injection at home	14.0	5.7	80.3
Monthly injection at a medical facility	16.0	7.0	76.9
Daily injection at home	21.7	8.1	70.2
Weekly injection at a medical facility	37.7	7.8	54.5
Daily injection at a medical facility	50.1	8.0	41.9

**Table 5:** Ranked responses by tolerance for side effects from a new therapy or treatment.

Possible treatment side effect	Some intolerance (%)	Neutral (%)	Some tolerance (%)
Temporary injection site pain	11.9	7.1	81.0
Mild skin irritation (slight redness)	13.4	7.6	79.0
Temporary allergic reaction at injection site	24.7	10.8	64.5
Moderate skin irritations (redness with swelling/itching)	24.8	11.4	63.7
Mild headache	29.9	13.4	56.6
Mild upset stomach	34.8	14.3	51.0
Mild nausea	43.3	14.4	42.2
Moderate headache	47.1	13.7	39.3
Mild joint pain	50.5	11.1	38.3
Moderate upset stomach	51.5	12.8	35.7
Mild chills/shaking chills	52.2	12.7	35.2
Moderate nausea	56.7	12.3	31.1
Moderate chills/shaking chills	60.2	11.4	28.4
Moderate joint pain	64.0	9.1	26.9
Severe allergic reaction that requires medical attention	88.7	5.0	6.3
Risk of internal bleeding	92.2	3.3	4.6
